# Triple therapy for neovascular age-related macular degeneration using single-session photodynamic therapy combined with intravitreal bevacizumab and triamcinolone

**DOI:** 10.1136/bjo.2008.150987

**Published:** 2009-03-08

**Authors:** P P Yip, C F Woo, H H Y Tang, C K Ho

**Affiliations:** 1 Department of Ophthalmology, Tuen Mun Hosptial, New Territories West, Cluster, Hong Kong; 2 The Hong Kong Ophthalmic Associates, Hong Kong

## Abstract

**Aim:**

To evaluate the efficacy and safety of triple therapy consisting single-session photodynamic therapy (PDT), intravitreal bevacizumab (IVB) and intravitreal triamcinolone (IVTA) for treatment of neovascular age-related macular degeneration (AMD)

**Methods:**

Consecutive patients with subfoveal choroidal neovascularisation (CNV) secondary to AMD were treated with PDT using a standard protocol immediately followed by 1.25 mg of IVB and 4 mg of IVTA. 1.25 mg of IVB was given at 3 months for residual leakage. Best-corrected Snellen visual acuity (BCVA) and fluorescein angiography (FA) were performed prior to treatment. BCVA, intraocular pressure (IOP) and presence of vitritis were documented at 1 and 6 weeks, 3 and 6 months. FA was repeated at 3 and 6 months. Outcome measures included visual improvement measured by logMAR equivalent, angiographic evident of leakage and safety profile.

**Results:**

36 eyes of 33 patients, aged 76.4 (SD 10.5) years with mean follow-up of 14.7 (6.9–19.2) months were analysed. Baseline logMAR acuity was 1.22 (0.71). The mean logMAR acuity was 1.14 (0.62) and 1.18 (0.63) at 3 and 6 months respectively. At 6 months, 61.1% (22/36) showed stable or gaining vision, and 27.8% (10/36) gained three or more lines. Twenty-eight eyes (77.8%) achieved CNV resolution by single session of triple therapy. One eye lost more than six lines due to retinal pigment epithelium rip, three eyes showed a significant cataract requiring surgery, and two showed persistent raised IOP at 6 months. None resulted in endophthalmitis or reported thromboembolic event.

**Conclusions:**

Short-term results of single session triple therapy suggested that it might be a useful treatment option for neovascular AMD based on its low retreatment rates, sustainable CNV eradication result and visual gain achievement. However, the risk and benefits of using intravitreal triamcinolone in addition to combined PDT and IVB warrant further evaluation.

Age-related macular degeneration (AMD) is one of the leading causes of blindness in the developed world. It can be classified into non-neovascular (dry) and neovascular (wet) form. The neovascular form of AMD is characterised by the development of choroidal neovascularisation (CNV). It contributed to a minority of cases, approximately 10% to 20% but associated with 80% to 90% of visual loss.^1^ It is clear that no single therapy addresses the multifactorial pathogenesis of the disease. In CNV, tissue ischaemia and/or inflammation from age-related changes triggers angiogenic signal molecules such as vascular endothelial growth factor (VEGF). Thus, the ideal therapeutic goal should achieve not only CNV eradication but also inflammation reduction and VEGF downregulation in order to produce sustainable effect.

Photodynamic therapy (PDT) with verteporfin works with its selective angio-occlusive effect. Its effectiveness varies among different types of CNV. According to the Treatment of Age-Related Macular Degeneration with Photodynamic Therapy study, it is most effective in predominantly classic type of CNV (at least half of the lesion is classic) in reducing the risk of visual loss.^2^ ^3^ In the Verteporfin in Photodynamic therapy report study, small, active, minimally classic or occult CNV lesions may also respond.^2^^–^4 However, PDT monotherapy mainly achieves visual stabilisation rather than visual improvement.^2^ ^3^ ^5^ Another drawback of PDT is the need for repeated treatments resulting from the high recurrence rate of CNV,^2^ ^3^ which compromises the success of the therapy.

Subsequently, anti-inflammatory agents, like intravitreal triamicinolone, have been introduced as an adjunct for PDT to limit further VEGF upregulation initiated by the therapy. This combination therapy has shown to be beneficial when compared with PDT monotherapy^6^^–^9 in terms of functional results and an extended treatment durability.^10^ ^11^

Antivascular endothelial growth factor (anti-VEGF), on the other hand, functions by blockade of VEGF-A, which is overexpressed in the process of the disease. There are a few commercially available anti-VEGF. Bevacizumab (Avastin) is a full-length anti-VEGF antibody which is approved for intravenous use in metastatic colon cancer.^12^ ^13^ Off-label intravitreal use has been shown to be effective in treating neovascular AMD.^14^ Ranibizumab (Lucentis) is an anti-VEGF fragment that binds all isoforms of VEGF-A.^12^ Pegaptanib sodium (Macugen), the first FDA approved anti-VEGF for intravitreal use, is an RNA aptamer that binds only VEGF-A isoform 165. Studies have shown enlightening results in achieving visual gain, including minimally classic or occult without classic neovascular AMD, which traditionally might not respond well to PDT.^15^ However, the action of anti-VEGF therapies seems to be transient. It inhibits continued neovascularisation but does not destroy existing CNV. As frequent retreatment is needed, this inevitably exposes patients to repeated risks of intravitreal injection, namely endophthalmitis and posterior segment complications.

Studies have been performed on single and combination use of these agents. Combination use of PDT and anti-VEGF has been shown to be superior to using either agent alone.^16^ ^17^ The purpose of this case series is to study the effectiveness of single session of triple therapy, namely photodynamic therapy, intravitreal injection of bevacizumab(avastin) and triamcinolone in management of neovascular AMD. We mainly focus on the anatomical, functional outcome and safety profile of single session of this therapy.

## METHODS

Consecutive cases of subfoveal choroidal neovascular (CNV) due to age-related macular degeneration were recruited from 19 December 2005 to 5 February 2007. Patients with fluorescent-angiography-diagnosed active subfoveal CNV due to age-related macular degeneration with minimum follow-up of 6 months were included. Patients with coexist pathology causing subfoveal choroidal neovascular such as myopic CNV, retinal angiomatosis proliferation, intraocular surgery performed during the study period or history of thromboembolic event were excluded.

Complete ophthalmic examinations including best-corrected Snellen visual acuity and intraocular pressure were recorded, and fluorescent angiography was performed in the first visit. Patients were then given photodynamic therapy with verteporfin using standard protocol (6 mg/m^2^, 50 J/cm^2^, 600 mW/cm^2^, 83 s), followed by intravitreal injection of 1.25 mg of bevacizumab (Avastin, Genentech) in 0.05 ml and 4 mg of filtered triamcinolone (Kenacort A, Bristol- Myers Squibb (HK)) in 0.1 ml within 1 h of PDT under aseptic conditions with paracentesis performed at the same time.

Patients were followed up 1 week, 6 weeks, 3 months and 6 months after treatment. Best-corrected Snellen visual acuity, intraocular pressure and presence of vitritis were recorded in every visit. Fluorescent angiographies were repeated at 3 and 6 months. Intravitreal bevacizumab (1.25 mg) was injected if fluorescent angiography showed features of persistent choroidal neovascularisation at 3 months. The above procedures and examinations were performed by a single surgeon in a single centre after informed consent.

A paired t test was used to compare findings before and after treatment. The Snellen visual acuity measured was converted to logMAR equivalent for statistical analysis.

## RESULTS

A total of 36 eyes of 36 patients were included in the analyses. The mean age was 76.4 years old; 66% were male. Right and left eye involvement was similar. Baseline lesions were 19.5%, 55.5% and 25% for predominantly classic, pure occult and minimally classic respectively; 58.3% were treatment-naive. The baseline logMAR visual acuity was 1.22, and the mean follow-up time was 14.7 months (table 1).

**Table 1:** Demographics of patients included in the study

Mean age in years (SD), range	76.4 (10.5), 52 to 92
Gender (n)	Male: 24; female: 12
Laterality (n)	Right: 19; left: 17
Prior treatment (n)	Naïve: 21; past treatment: 15
Baseline lesion types (n)	Predominantly classic: 7 (19.5%)
	Pure occult: 20 (55.5%)
	Minimally classic: 9 (25%)
Mean (SD) follow-up, range	14.7 (2.9), 6.9 to 19.2 months

The outcome analysis focused on functional outcome, anatomical outcome and evaluation of the safety of treatment.

**Figure 1:**
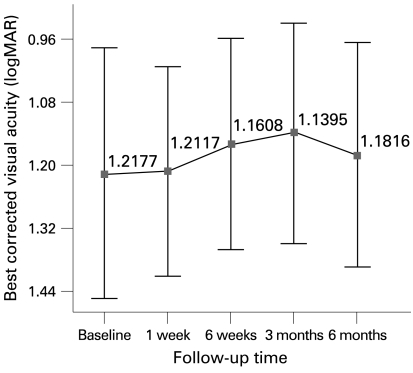
Change in best-corrected visual acuity form baseline to 6 months follow-up after triple therapy. The grey squares joined together with lines show the means. Error bars show the 95% CI of the mean.

Functional outcome was defined as the change in visual acuity from baseline using logMAR equivalent. After a single session of triple therapy, 66.7% and 61.1% achieved stable or gaining vision 3 and 6 months, respectively. However, the percentage with loss of six or more lines was 5.6% at 3 months, which increased to 13.9% at 6 months (table 2). Longitudinal analysis suggested a trend of visual improvement with a maximum effect at 3 months (p = 0.323) (fig 1). A subgroup analysis for the naive group versus the pretreatment group suggested insignificant results.

**Table 2:** Visual acuity distribution after single session of triple therapy for neovascular age-related macular degeneration

LogMAR best-corrected visual acuity	3 months (%) (n)	6 months (%) (n)
Gaining vision	52.7 (19/36)	41.7 (15/36)
⩾3 lines	30.5 (11/36)	27.8 (10/36)
<3 lines	22.2 (8/36)	13.8 (5/36)
Stable vision	13.8 (5/36)	19.4 (7/36)
Losing vision		
<3 lines	8.3 (3/36)	13.9 (5/36)
⩾3 to <6 lines	13.9 (5/36)	8.3 (3/36)
⩾6 lines	11.1 (4/36)	16.7 (6/36)

Anatomical outcome was defined as complete resolution of angiographic evidence of CNV. After a single session of triple therapy, 28 of 36 eyes (77.8%) achieved angiographic resolution. There were persistent CNV in eight of 36 eyes (22.2%). The percentage of anatomical outcome was the same at 3 and 6 months.

Regarding treatment safety, three (8.3%) had a significant cataract requiring surgery, and one (2.8%) lost more than six lines due to retinal pigment epithelium rip. None had pigment epithelial detachment. There were nine (25%) cases observed with mild vitritis in the first 6 weeks, but all subsided spontaneously. There were one transient ocular hypertension in the first week and four at 6 weeks, all resolving with topical medication. There were two cases encountered with persistent elevated intraocular pressure requiring treatment for 6 months. Neither endophthalmitis nor a thrombo-embolic event was reported in this series.

**Figure 2:**
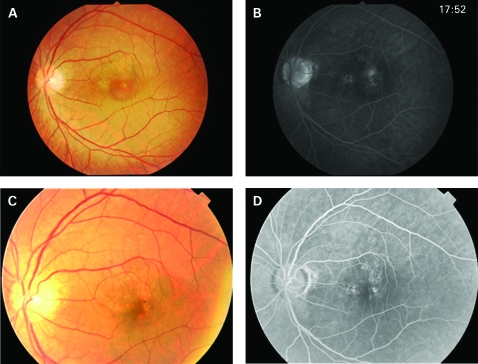
Fundus and fluorescein angiography images of a case receiving triple therapy.

Figure 2 shows fundus and FA images of a case receiving triple therapy. On presentation (fig 2A, B), the left-eye visual acuity was 0.5, and FA showed occult CNV. At month 3 after treatment (fig 2C, D), the VA improved to 0.8, and CNV dried up. Results were sustained at month 6. This case illustrated successful eradication of CNV by a single session of triple therapy.

**Figure 3:**
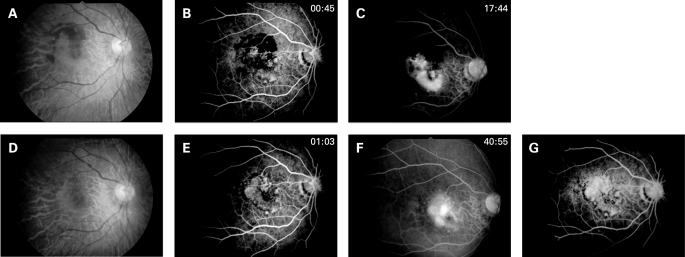
Fundus and fluorescein angiography images of a case receiving triple therapy with supplementary bevacizumab at month 3.

Figure 3 shows fundus and FA images of another case receiving triple therapy with supplementary bevacizumab at month 3. On presentation (fig 3A–C), the right-eye visual acuity was 0.2. FA showed large occult CNV with late leakage. At month 3 (fig 3D–F), VA was 0.05, and evidence of persistent angiographic leakage was found. Intravitreal bevacizumab (fig 3G) was given, and FA repeated at month 6 showed a significant reduction in angiographic leakage. The VA improved to 0.16, and the size of the scotoma was reduced. Although this case did not achieve CNV eradication, visual function was improved. This illustrated the beneficial effect of triple therapy in reducing the area and extent of leakage.

## DISCUSSION

Our study showed that a single session of triple therapy using a standard protocol of PDT by verteporfin, intravitreal bevacizumab and triamcinolone in treating neovascular AMD yielded encouraging results. In our series, 78% achieved CNV eradication, and 61% achieved visual stabilisation at 6 months, with 30% and 28% gaining three or more lines at 3 and 6 months respectively. Successful CNV eradication at 3 months remained so for 6 months. Therefore, a single session of triple therapy is indeed time-saving with sustainable effects.

In a randomised controlled study performed in Vienna, Austria comparing 1 mg of intravitreal bevacizumab with the combination of standard PDT by verteporfin and 4 mg of IVTA for neovascular AMD, the use of intravitreal bevacizumab alone appears to result in significantly better visual outcomes.^18^ However, at 3 and 6 months, 50% and 43% required retreatment in the bevacizumab group, while 79% and 14% required retreatment in the combination group. This finding supported the fact that PDT plus IVTA achieved better results in CNV eradication at 6 months, but the functional outcome is less promising when compared with intravitreal bevacizumab. In our single-session triple therapy, only 22% and 22% required retreatment at 3 and 6 months respectively. Therefore, triple therapy may have a role in reducing the retreatment rate while preserving the functional benefit of bevacizumab.

Other regimens of triple therapy in neovascular AMD are recently published, and they all showed a significant functional improvement. In the series by Augustin *et al* which included 104 eyes, reduced light dose of PDT by verteporfin, 800 μg of dexamethasone and 1.5 mg of bevacizumab intravitreal injection were used.^19^ After one cycle of treatment, the mean visual acuity was significantly improved by 1.8 lines, with 39.4% gaining three or more lines and 3.8% losing three or more lines. In another series by Ahmadieh *et al*, which included 17 eyes, a standard PDT protocol, 1.25 mg of bevacizumab and 2 mg of triamcinolone intravitreal injection, was used.^20^ The mean pretreatment BCVA was 0.74 (0.33) (logMAR) which was significantly improved to 0.52 (0.36) at 3 months. Our triple therapy series demonstrated a trend of visual improvement with a mean of 0.78 lines at 3 months. However, the functional improvement is less obvious at 6 months. Possible reasons included:

- The use of 4 mg IVTA. The higher dosage of IVTA increases the risk of side effect, mainly cataract, vitritis and ocular hypertension. These accounted for the proportion of visual lost in our series.
- Eyes that were previously treated by PDT were not excluded. As multiple PDT regimens may have an adverse effect on physiological choroids, RPE and even neurosensory retina,^21^ ^22^ visual improvement may be underestimated or even limited by our suggested triple therapy.

In contrast, in the series by Ahmadieh *et al*, 58.8%, versus 22% in our series, required a second injection at 3 months. This may be attributed to our higher dosage of IVTA used. The latter questions will remain to be investigated by further comparative studies with adjusted dosage of IVTA.

A single session of triple therapy, therefore, has its potential advantages. It reduces the risk of multiple PDT, which may induce CNV recurrence by aggravation of choroidal ischaemia and subsequent overexpression of VEGF.^23^ Triamcinolone has been shown to suppress the early proangiogenic response in RPE cells after PDT in vitro^24^ and itself affects CNV independently.^25^ Intravitreal bevacizumab can work synergistically with PDT against any existing CNV and also counteract the adverse effect of PDT. Thus, triple therapy saves treatment costs, consultation time and unnecessary multiple intravitreal injections.

Since the era of PDT, plenty of articles have been published on the combined use of PDT with intravitreal triamcinolone. Our trial was aimed at studying the additional effect of an extra agent, bevacizumab, in managing neovascular AMD. It is not until recently, in 2007, another regimen of triple therapy, that replacing triamcinolone by dexamethasone has been advocated.^19^ It is believed that the shorter half life of dexamethasone with its additional antifibrotic, antiproliferative and antimigration properties can result in a better efficacy with fewer side effects than with triamcinolone. However, there are few published papers on the use of combined PDT with dexamethasone before and during our study. We believe that the use of dexamethasone may result in fewer side effects, mainly raising intraocular pressure; however, its efficacy may need to be proven by a larger trial.

Our study is not without its limitations. Supplementary information of retinal thickness would be useful to show the extent and effect of therapy in the reduction of macular oedema. The number of CNV subtypes and previously treated group versus non-treated eyes were too small for a meaningful subgroup analysis. The intrinsic limitation of case series precluded statistically significant results, so only a trend of visual improvement was shown in the results of functional outcome. Therefore, a larger-scale comparative study with optical coherent tomography findings and use of different doses and types of anti-inflammatory or antiangiogenic agents would be helpful to provide further information on the best treatment regimen for neovascular CNV in age-related macular degeneration.

## CONCLUSION

Short-term results of single-session triple therapy suggest that it might be a useful treatment option for neovascular AMD based on its low retreatment rates, sustainable CNV eradication result and visual-gain achievement. However, the risks and benefits of using intravitreal triamcinolone, in comparison with other anti-inflammatory agents, in triple therapy warrant further evaluation.
